# Effect of Spinal Cord Stimulation on Gait in a Patient with Thalamic Pain

**DOI:** 10.1155/2016/8730984

**Published:** 2016-08-07

**Authors:** Arito Yozu, Masahiko Sumitani, Masahiro Shin, Kazuhiko Ishi, Michihiro Osumi, Junji Katsuhira, Ryosuke Chiba, Nobuhiko Haga

**Affiliations:** ^1^Department of Rehabilitation Medicine, The University of Tokyo Hospital, 7-3-1 Hongo, Bunkyo-ku, Tokyo 113-8655, Japan; ^2^Department of Pain and Palliative Medicine, The University of Tokyo Hospital, 7-3-1 Hongo, Bunkyo-ku, Tokyo 113-8655, Japan; ^3^Department of Neurosurgery, The University of Tokyo Hospital, 7-3-1 Hongo, Bunkyo-ku, Tokyo 113-8655, Japan; ^4^Neurorehabilitation Research Center, Kio University, 4-2-2 Umaminaka, Koryo-cho, Kitakatsuragi-gun, Nara 635-0832, Japan; ^5^Department of Prosthetics and Orthotics and Assistive Technology, Faculty of Medical Technology, Niigata University of Health and Welfare, 1398 Shimami-cho, Kita-ku, Niigata, Niigata 950-3198, Japan; ^6^Research Center for Brain Function and Medical Engineering, Asahikawa Medical University, 1-1-1 Higashi-2-jyou, Midorigaoka, Asahikawa, Hokkaido 078-8510, Japan

## Abstract

Thalamic pain is a central neuropathic pain disorder which occurs after stroke. Its severe chronic pain is often intractable to pharmacotherapies and affects the patients' activities of daily living (ADL) and quality of life (QOL). Recently, spinal cord stimulation (SCS) has been reported to be effective in relieving the pain of thalamic pain; however, the effect of SCS on gait performance in patients is unknown. Therefore, we evaluated the gait performance before and after SCS in a case with thalamic pain. A 73-year-old male with thalamic pain participated in this study. We evaluated the gait of the patient two times: before SCS insertion and after 6 days of SCS. At the second evaluation, we measured the gait in three conditions: stimulation off, comfortable stimulation, and strong stimulation. SCS succeeded in improving the pain from 7 to 2 on an 11-point numerical rating scale. Step frequency and the velocity of gait tended to increase between pre- and poststimulation periods. There were no apparent differences in gait among the three stimulation conditions (off, comfortable, and strong) at the poststimulation period. SCS may be effective on gait in patients with thalamic pain.

## 1. Introduction 

Thalamic pain is a central neuropathic pain disorder, which occurs after thalamic stroke. Of all the stroke survivors, 2.7%–8% patients suffer from thalamic pain [1, 2]. They generally experience severe chronic pain in the hemibody opposite to the thalamic lesion, and thalamic pain is often intractable to various pharmacotherapies. Consequently, thalamic pain generally affects the activities of daily living (ADL) and quality of life (QOL) in patients after stroke [3].

Spinal cord stimulation (SCS) is used to treat intractable pain disorders, including both central and peripheral neuropathic pain. For peripheral neuropathic pain, SCS has proven to be effective in relieving pain [4–8]. For thalamic pain (central neuropathic pain), the analgesic effect of SCS used to be uncertain; however, two recent studies showed its effectiveness in patients with central neuropathic pain [9, 10]. Lopez et al. reported that pain relief was satisfactory in 6 of 8 patients [10]. Aly et al. reported that half of the 30 patients experienced good or fair pain relief during the SCS trial [9].

Considering the clinical usefulness of SCS in patients with thalamic pain, we should focus not only on pain relief but also on its effect on gait because gait performance is the critical element of ADL and has a heavy impact on QOL [11–15]. Rijken et al. examined the effect of SCS on gait in peripheral neuropathic pain and found no significant change in step frequency, velocity, and step length [16]. However, there is no study that examined the effect of SCS on gait in central neuropathic pain. Therefore, how gait performance would change by SCS in central neuropathic pain is unknown. We may expect an increase in gait performance due to the pain relief. In this study, we first evaluated the gait performance before and after SCS in a single patient with thalamic pain.

## 2. Subject and Methods 

### 2.1. Patient

A 73-year-old male with thalamic pain in his right hemibody for 2 years participated in this study. After left thalamic hemorrhage, he suffered from right hemiparesis and poststroke thalamic pain, mainly in his right upper and lower extremities. Thalamic pain was resistant to some pharmacotherapies, including pregabalin, antidepressants, and opioid, and he subsequently received SCS treatment. The patient provided his written informed consent prior to the study. All the procedures were performed in accordance with the Declaration of Helsinki, and they were approved by the institutional ethics committee.

### 2.2. SCS Procedure

The operation for SCS lead insertion was performed under local anesthesia with the guidance of fluoroscopy. Two eight-electrode leads (Octrode; St. Jude Medical, Inc., St. Paul, Minnesota, USA) were implanted at the 5th cervical (C5) and 8th thoracic (Th8) vertebrae-level epidural space. The upper lead treated pain in the upper extremity and the lower in the lower extremity. The leads were connected to a pulse generator (Genesis Patient Programmer; St. Jude Medical, Inc., St. Paul, Minnesota, USA).

Stimulation was initiated 1 day after the operation. A daily stimulation protocol was set at the discretion of the patient. Basically, the patient switched on the SCS generator during daytime and switched it off when he was asleep. When it was switched on, the amplitude was set between 0.8 and 8.0 mA, a rate of 4 Hz, and pulse width with 210–300 *μ*sec. The stimulation protocol was selected by himself to induce the distribution of  “comfortable” sense to his upper and lower extremities.

### 2.3. Pain Assessment

We used an 11-point numerical rating scale (NRS) for pain assessment [17–20]. The NRS is a segmented numerical version of the visual analog scale. The most commonly used is the 11-item NRS, in which the patient selects a whole number (0–10) that best reflects the intensity of his pain [17–20]. Here, 0 represents “no pain” and 10 represents “pain as bad as you can imagine.”

### 2.4. Gait Assessment

**Figure 1:**
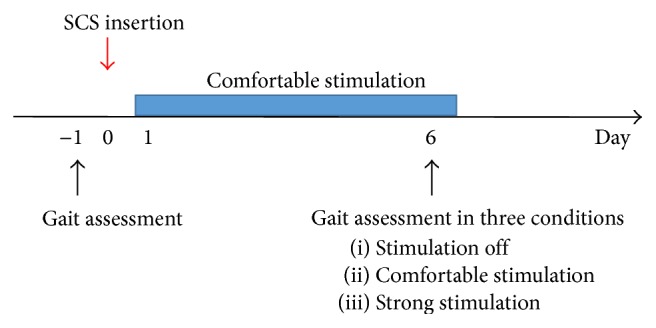
The study design. We evaluated the gait two times: the day before spinal cord stimulation (SCS) insertion and after 6 days of SCS period. At the second evaluation, we measured three conditions: SCS off, SCS comfortable, and SCS strong.

We evaluated the gait of the patient two times: the day before SCS insertion and after 6 days of SCS period (Figure 1). His gait was measured by the motion analysis system (VICON MX; VICON Motion Systems Ltd., Oxford, UK). Reflective markers were placed on the body according to the modified Helen Hayes marker set [21]. Spatiotemporal parameters (step frequency, velocity, and stride length) and kinematics (range of motion (ROM) in extension/flexion of the hip, knee, and ankle during the gait) were calculated.

At the first evaluation, we could collect nine gait cycles from three trials. This was our best effort before the patient became too tired to continue. At the second evaluation, we measured the gait in three conditions: stimulation off, stimulating the SCS lead for the lower extremity under a “comfortable” setting, and stimulating under a “strong (but not noxious)” setting. We measured the gait of each condition immediately after switching to each condition during the second evaluation. The comfortable setting showed the amplitude of 3 mA for the lower SCS lead, and the strong setting showed 5 mA. Using 5 mA amplitude stimulation, he experienced a slight muscle twitch in his right foot. To extract the effect of SCS in the lower extremity, we did not stimulate the upper SCS lead for the upper extremity in any of these three conditions. In these three conditions of the second evaluation, we could collect 12 gait cycles from four trials for each condition. In total, it took 16 minutes to measure the three conditions.

### 2.5. Statistical Analysis

In total, we measured the gait in four conditions: (1) pre-SCS insertion, (2) stimulation off after 6 days of the SCS period, (3) “comfortable” stimulation after 6 days of the SCS period, and (4) “strong” stimulation after 6 days of the SCS period. We compared the spatiotemporal parameters and kinematics of these conditions using one-way analysis of variance (ANOVA). We set the significant level strictly at *p* < 0.01 to avoid false positives because of a single-case study. Multiple comparisons were made with the use of the Bonferroni method. Statistical analyses were performed using the Statistical Package for the Social Sciences (SPSS) (ver. 22, IBM Corp., NY, USA).

## 3. Results

### 3.1. Pain

The pain existed in the patient's whole right lower limb (from the toes to the hip and inguinal region) and in his right hand. The pain was 7 on the NRS before SCS and improved to 2 after 6 days of SCS. Because of the residual effect of SCS, the pain did not deteriorate even under the SCS-off condition in the post-SCS measurement.

### 3.2. Spatial and Temporal Parameters

**Figure 2:**
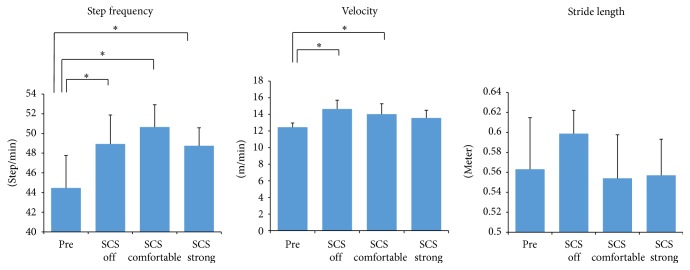
Mean values for step frequency, velocity, and stride length of the gait. The error bars represent one standard deviation. The asterisks indicate significant difference (*p* < 0.01).

The measurements of step frequency, velocity, and stride length in four conditions are shown in Figure 2. The step frequency significantly increased at the three postperiod conditions compared with that at the preperiod. There were no significant differences among the three poststimulation period conditions with respect to the step frequency.

The velocity of gait increased significantly between preperiod and SCS off at the postperiod and between preperiod and comfortable SCS at the postperiod. There were no significant differences among the three postperiod conditions with respect to the velocity.

Further, there were no differences in the stride length among the four conditions.

### 3.3. Kinematics

ROM in extension/flexion during the gait in the lower extremities is shown in Table 1. The motion of the left hip increased significantly between the prestimulation period and the other three poststimulation period conditions. However, the other remaining joints did not show any apparent change after 6 days of the SCS period.

## 4. Discussion

This study is the first to report the effect of SCS on gait in a patient with thalamic pain. SCS succeeded in improving the pain from 7 to 2 on an 11-point NRS. Step frequency and the velocity of gait tended to increase between pre- and poststimulation periods. There were no apparent differences in gait among the three stimulation conditions (off, comfortable, and strong) at the poststimulation period.

### 4.1. Pre- versus Poststimulation Period

The velocity of the gait tended to increase at the poststimulation period. Because velocity is the multiplication of length by frequency and because the stride length did not differ apparently between the conditions, the increase in velocity is due to the increase in step frequency.

The result of our study was different from that of Rijken et al. [16], which examined the effect of SCS in peripheral neuropathic pain and found no significant change in step frequency or velocity. Such a difference may exist on the original motor performances of the participants and also on methodological issues. The participants of the previous study had moderate gait impairment (e.g., the velocity was approximately 50 m/min), whereas our participant had more severe gait impairment (e.g., the velocity was approximately 14 m/min), and the impact of SCS did not appear in the previous study. Furthermore, the previous study used a treadmill to measure the gait performance. Generally, in treadmill walking, participants must adapt to the belt; therefore, measurements obtained by a treadmill are not necessarily similar to their natural gait performance. On the other hand, our study examined level gait, which would reflect more natural motor performance.

One most possible mechanism for the increase of the frequency is that the pain relief permitted the patient to move more freely. An intimate relationship between pain and motor dysfunction is known. For example, a study on patients with intermittent claudication demonstrated that the pain was related to the impairment of step frequency and walking speed [22]. This pattern is similar to our study's pattern.

The gait performance was improved even in the SCS-off condition compared with the pre-SCS condition. SCS has a residual effect on pain relief, and the patient did not complain of pain deterioration even when the SCS was turned off at post-SCS measurement. Compared with the pre-SCS condition, the patient had less pain in the SCS-off condition. We think this is why the gait improved in the SCS-off condition compared with the pre-SCS measurement.

Despite pain being present in the right hemibody, which was relieved by SCS, the joint motion increased in the left hip and not in the right. This is not surprising because the right hip is also the side of hemiparesis, and the effect of the pain relief might not be seen. On the other hand, the left hip is a proximal joint and adjacent to the right. Therefore, SCS may release the left hip from its restriction caused by the surrounding pain.

Another possibility for gait restoration is the effect of SCS apart from pain relief. There are studies on Parkinson's disease, spinal cord injury, and ataxia, where gait was improved by SCS [23–27]. The mechanism for this effect is unclear. One proposed is that locomotion is increased by the disruption of antikinetic oscillatory synchronization in the corticobasal ganglia circuits through the activation of lemniscal and brainstem pathways [24].

### 4.2. Poststimulation Period

There were no apparent differences among three stimulating conditions at the poststimulation period. SCS stimulates the dorsal column of the spine, including the medial lemniscal tract, which is the pathway of proprioception. Thus, SCS may interfere with proprioception and might affect the gait [28]. However, our data showed no apparent change even in strong stimulation. We tested level gait in our study, whereas dual-task gait or eye-closed-balance test may extract the effect on proprioception more sensitively. This will be our future work.

### 4.3. Limitations

This study examined only a single subject. Furthermore, we only studied one point after the insertion of SCS. The patient might adapt to SCS, and gait performance may change in the long term. A group study with a long-term follow-up is needed to evaluate the definite effect of SCS on gait in patients with thalamic pain. Nonetheless, this was the first study to highlight the gait performance in central neuropathic pain after SCS.

## Figures and Tables

**Table 1:** Range of motion in extension/flexion during gait.

	Pre-SCS		SCS off		SCS comfortable		SCS strong	
	Mean	SD	Mean	SD	Mean	SD	Mean	SD
Left hip	31.8	2.1	37.5	2.6^**∗**^	35.8	1.7^**∗**^	36.4	2.8^**∗**^
Right hip	31.0	6.3	33.4	3.4	31.4	2.4	30.9	3.5
Left knee	46.7	3.6	48.2	2.5	47.8	1.9	48.2	2.0
Right knee	41.8	11.4	37.7	5.8	34.4	2.9	35.3	7.3
Left ankle	25.0	4.8	29.9	4.8	30.9	2.6	29.6	4.0
Right ankle	11.4	2.8	13.4	2.1	13.0	1.5	13.4	4.2

^*∗*^ *p* < 0.01; significant difference between pre-SCS and SCS off, between pre-SCS and SCS comfortable, and between pre-SCS and SCS strong.

SCS, spinal cord stimulation.

SD, standard deviation.
